# The Effects of Adopting Mobile Health and Fitness Apps on Hospital Visits: Quasi-Experimental Study

**DOI:** 10.2196/45681

**Published:** 2023-07-28

**Authors:** Yan Bo, Qianqian Ben Liu, Yu Tong

**Affiliations:** 1 Department of Data Science and Engineering Management School of Management Zhejiang University Hangzhou China; 2 Department of Information Systems College of Business City University of Hong Kong Hong Kong China; 3 Center for Research on Zhejiang Digital Development and Governance Hangzhou China

**Keywords:** health and fitness apps, app adoption, app use, hospital visit, causal effect, health behavior, consumption level, city tier, digital literacy, hospitalization, admission, adoption, acceptance, mobile health, mHealth, fitness, exercise, physical activity, health app, fitness app, difference-in-differences

## Abstract

**Background:**

Overcrowding in public hospitals, a common issue in many countries, leads to a range of negative outcomes, such as insufficient access to medical services and patient dissatisfaction. Prior literature regarding solutions to reducing hospital overcrowding primarily focuses on organizational-level operational efficiency. However, few studies have investigated the strategies from the individual patient perspective. Specifically, we considered using mobile health and fitness apps to promote users’ health behaviors and produce health benefits, thereby reducing hospital visits.

**Objective:**

This study estimated the causal effect of health and fitness app adoption on hospital visits by exploiting the staggered timing of adoption. We also investigated how the effect varied with users’ socioeconomic status and digital literacy. This study provides causal evidence for the effects of health apps, extends the digital health literature, and sheds light on mobile health policies.

**Methods:**

This study used a data set containing health and fitness app use and hospital-related geolocation data of 267,651 Chinese mobile phone users from January to December 2019. We used the difference-in-differences and difference-in-difference-in-differences designs to estimate the causal effect. We performed a sensitivity analysis to establish the robustness of the findings. We also conducted heterogeneity analyses based on the interactions of postadoption indicators with users’ consumption levels, city tiers, and digital literacy.

**Results:**

The preferred model (difference-in-difference-in-differences) showed a significant decrease in hospital visits after the adoption of health and fitness apps. App adoption led to a 5.8% (*P*<.001), 13.1% (*P*<.001), and 18.4% reduction (*P*<.001) in hospital visits 1, 2, and 3 months after adoption, respectively. In addition, the moderation analysis shows that the effect is greater for users with high consumption levels, in high-tier cities, or with high digital literacy.

**Conclusions:**

This study estimated the causal effect of health and fitness app adoption on hospital visits. The results and sensitivity analysis showed that app adoption can reduce users’ hospital visits. The effect varies with users’ consumption levels, city tiers, and digital literacy. These findings provide useful insights for multiple stakeholders in the Chinese health care context.

## Introduction

Public hospitals in many countries are in a chronic overcrowding crisis. Overloaded public hospitals are common, especially in low-income countries. For example, in many Chinese public hospitals, the typical daily inpatient count exceeds twice the number of inpatient beds [1]. Overcrowding creates substantially adverse effects on both patients and health professionals. It leads to insufficient access to health care, difficulties in making appointments, and long wait times [2]. It also causes fatigue, burnout, and frustration among medical staff [3]. These may further lead to medical errors, patient dissatisfaction, and medical disputes [1,4]. On the other hand, the growing health care costs decrease the use of health care resources [5]. Against this background, academics and health care providers have become increasingly interested in investigating other factors that may affect health care use. As an essential form of health care use, hospital visits may generate substantial economic and social impacts. Therefore, it is important to investigate the factors that impact hospital visits and probe into solutions to overcrowding.

Recent studies on overcrowding in emergency departments point to the continuously increasing demand for health services as a significant cause and suggest organizational-level strategies to improve operational efficiency and patient flow in hospitals [6,7]. For example, team triage is a typical strategy to enable a team to perform an effective initial assessment of patients. It can reduce the likelihood of patients entering a wrong department and leaving without being treated [8], thereby improving patient flow and alleviating the problem of overcrowding. Various models have also been developed to optimize scheduling in operating rooms and emergency departments [9,10]. Nevertheless, few studies have examined the options for reducing hospital visits from the individual patient perspective. It is believed that improving people’s health is a fundamental way to reduce the demand for health services.

Among the means of improving people’s health, preventive health behaviors (eg, exercises, proper eating habits, and healthy lifestyles) can reduce the risk of most chronic diseases and generate significant health benefits [11,12]. In recent years, there has been robust growth in *mobile health (mHealth) and fitness apps*. These apps seek to help people adopt a healthy lifestyle [13]. They typically contain features to record and monitor users’ health metrics (eg, heart rate and sleep time) and exercises, provide diet and exercise recommendations, schedule health behaviors and send reminders, and enable interactions with other users. They offer a new way to improve preventive health behaviors and can potentially play a role in improving the health status of the general population [14]. As such, they might be a low-cost solution for the overloaded public health systems.

However, health and fitness apps might also hinder the touted benefits [15]. First, adopting health and fitness apps may not guarantee continuous use. For example, 21% of users give up on the apps after using them only once [16]. Second, exercises without guidance or supervision could result in injuries (eg, skeletal muscle damage [17]), psychological issues such as overexercising, unhealthy diet habits (eg, obsession with healthy eating), or compulsive behaviors [18]. Third, the lack of policies that protect user privacy lowers trust in these apps [19,20], thus hindering the realization of health benefits [21].

The different arguments infer contradictory views regarding the effect of health apps. However, it is difficult to inform policy without systematic evidence of which account is more representative of reality. It is important to note that prior research on mHealth mainly focused on improvements in health indicators (eg, blood pressure [22] and weight loss [23]), behavioral changes (eg, physical activity [24] and diet [25]), and patient perceptions (eg, self-efficacy [26]) [27], rather than directly capturing hospital visits. Examining hospital visits, a substantial outcome of health behaviors, could offer direct implications on the issue of hospital overcrowding and help address the contradictory views in the literature.

This study assessed the impacts of mHealth and fitness app adoption on hospital visits. We leveraged a data set of anonymous mobile phone users in China, which contains these users’ installation and use of focal health and fitness apps and the duration of hospital visits calculated based on the users’ hospital-related geolocation data.

Our study has several advantages compared with prior studies. First, this research empirically identifies the causal effect of mHealth and fitness app adoption on health outcomes. Many existing studies have not established causal links between mHealth apps and health outcomes (and offline health services) [28,29], as their findings might be confounded by many unobservable factors (eg, people’s health consciousness and health status). Other findings may suffer from measurement errors stemming from self-reported data [30], which are susceptible to recall and social desirability biases. Our empirical strategy exploits the staggered timing of app adoption to address these causal inference challenges. Second, existing studies typically focus on specific population groups (eg, older adults [31] and college students [30]) or patients with specific diseases (eg, rheumatic diseases [32] and specific chronic health conditions [31]). Our study examined a broader group of mobile phone users, making the findings more generalizable. By combining causal inference strategies and big data, we extended the research paradigm in the field of medical informatics from randomized controlled trials and surveys to observational data. Third, going 1 step beyond the studies investigating factors that affect health app use [33-35], we showed that these factors also moderate the link between health app use and hospital visits. This may provide a more complete and connected view of the effects of mHealth and fitness apps on health outcomes.

## Methods

### Data

#### Overview

The data set used in this study was sourced from a leading Chinese mobile app platform (hereinafter referred to as the platform). The original data set comprises anonymized statistical and predicted data that are deidentified, filtered, cleaned, and organized using advanced data security and data mining techniques. The data set used in this study, which was randomly sampled from the original data set, contained random anonymous users’ focal health and fitness app use and hospital-related geolocation data from January to December 2019. The sampling period was chosen because it was before the outbreak of the COVID-19 pandemic, and no other public health shocks could confound the link between mobile app adoption and hospital visits.

Our data set is well suited to study the impact of mobile app adoption on hospital visits for several reasons. First, previous research shows that it is feasible to analyze the patterns of visits to medical facilities from the internet geotagged data [5]. Our data set contains hospital-related geolocation data, allowing us to explore the patterns of and factors influencing users’ hospital visits. Second, it contains information on focal app use. Although the data are deidentified and anonymized (hence no privacy concerns are involved), an anonymized identity of each item in the data set allows us to match the user’s app use with geolocation data. Third, the original data set of the platform was a representative sample of Chinese mobile app users. As our data set is a random sample of this original data set, we could generalize our findings to a wider population and investigate heterogeneous effects across various population groups.

#### Geolocation Data

Using geolocation data is an emerging way of constructing variables of interest in the health care context. As one specific form of digital phenotyping, which refers to the collection of data in real life via sensors, keyboards, and other features of mobile devices [36,37], geolocation data have been used to monitor and measure symptoms and behaviors in patients with various diseases (eg, schizophrenia [38] and depression [39]) and have been reported to be highly consistent with ecological momentary assessment reports of location [40].

The original geolocation data stream is split into 1-hour epochs, which contain hourly records of users’ hospital-related geographic locations. We used these data to operationalize our dependent variable, users’ hospital visits. This practice is consistent with the existing literature [41]. Technical issues (in tracking people’s locations), environmental factors (eg, weak mobile signals because of weather), or users’ behaviors (eg, turning off the GPS feature) may lead to measurement errors [41,42]. To reduce measurement errors, we counted a geolocation record as a hospital visit only when the user of that record stayed in a hospital for more than an hour (ie, for 2 consecutive epochs or more). We also excluded people who are physicians or web-based ride-hailing drivers (revealed by the platform’s big data model tags) when constructing the dependent variable because they may visit hospitals frequently for purposes that are not of interest to us. We aggregated the hourly records into monthly data, which results in our dependent variable indicating the number of hours user *i* is in a hospital in month *t* during our sampling period (ie, from January to December 2019).

#### Mobile App Use Data

We selected the most commonly used health and fitness apps in China. The selected app reminds users to engage in physical activity (eg, running, walking, and indoor training), helps record and manage their exercise status, and provides fitness guidance [43,44]. It is possible that users who adopt different categories of health and fitness apps could generate a synergistic effect. For instance, an exercise app and a diet app could promote the effects of each other. To rule out this confounding synergistic effect between different categories of apps, we also selected other commonly used representative health and fitness apps and excluded the users that adopted more than one category of apps.

**Figure 1:**
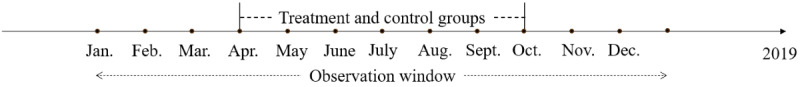
Timeline of sample data.

We focused on users who first installed the app from April to September 2019. The estimation relied on the different timing of adoption, constructing counterfactuals for app adopters by selecting users who adopted the same app but in other months within the sampling period. Specifically, the timing of health and fitness app adoption for the treatment and control group users was concentrated in the period from April to September 2019, but we expanded the observation window of users’ hospital visits to the period from January to December 2019. Thus, the period from January to March 2019 allows for an examination of preadoption trends, and the period from October to December 2019 allows for a meaningful identification of the postadoption effect on hospital visits. The timeline of our sample data is shown in Figure 1.

Our data also contain whether and when each user used the selected app. This allowed us to identify users who installed and used the app as well as those who installed but never used the app. As detailed in the next section, although the baseline estimation is based on all adopters, incorporating subgroups divided by their use in the analysis will help reduce bias in the estimation.

### Ethical Considerations

This study is exempt from human subject research ethics review for the following reasons. First, this study uses secondary, observational data sourced from a Chinese mobile app platform. The platform, under the users’ informed consent, collects observational data through partner mobile apps and constructs anonymized statistical and predicted data sets through modeling and data mining techniques. Privacy protection techniques (eg, pseudonymization) were used during data collection. Second, the data used in this study were anonymous and did not contain any personally identifiable information. Reidentification was also not possible from this data set. The data set was stored in specialized secure data servers, and the analysis process was handled at a site designated by the platform. The process was conducted for research purposes and complied with China’s data privacy laws and regulations. Third, this study does not involve experimental manipulations of human subjects or other ethical issues (eg, does not involve the data of children aged <18 years, which requires parental or guardian custody by law; does not involve sensitive aspects of the participants’ own behaviors; and does not pose any physical, psychological, or financial harm or risks to the participants in the study).

### Empirical Models

The empirical analysis sought to identify the causal effect of health and fitness app adoption on users’ hospital visits. The observed link between health and fitness app adoption and hospital visits may not have a causal interpretation because time-varying unobservable factors (eg, people’s health consciousness and health status) may be correlated with both app adoption and hospital visits and thus confound the relationship. If people adopt a health and fitness app entirely at random, it would generate an ideal experiment for estimating the effect of app adoption. People who did not adopt the app provided counterfactuals for people who adopted the app.

To approximate this experiment, we used a quasi-experimental design that uses the approximate randomness of app adoption within a short period. Specifically, we constructed counterfactuals for app adopters by selecting users who adopted the same app but in other months. Therefore, in our quasi-experimental design, for people in the treatment group who adopted the health and fitness app in a particular month, the control group comprised people from the same cohort who adopted the same app in other months within the sampling period. When the difference in the timing of app adoption is small, the treatment and control groups are more comparable, which means that the users are less likely to be systematically different in terms of their health status and health consciousness before the app adoption. This design removes the potential systematic differences in unobservable factors between adopters and nonadopters because both the treatment and control groups adopted the app. A similar research design was used in previous studies in the health care context [45,46].

We first performed an event study analysis, a commonly used method to analyze observational data in the health economics literature [47,48]. Specifically, we saturated the 2-way fixed effects difference-in-differences (DD) with the leads and lags of adoption time indicators, which leads to a dynamic DD specification. This specification estimates the effects in the preadoption and postadoption periods so that we can probe the parallel trends assumption. Adopting a health and fitness app may not cause an immediate and permanent change in health outcomes. It takes time to generate an effect and probably approach a new level. As the 2-way fixed effects DD estimator cannot unveil the dynamic pattern, we adopted a dynamic DD specification. The baseline specification is as follows:


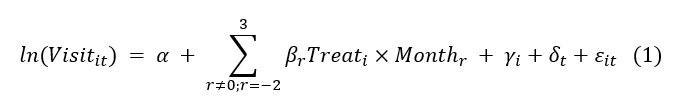


where *Visit_it_* denotes the duration of hospital visits for user *i* in month *t*; *Treat_i_* indicates whether a user belongs to the treatment group; *Month_r_* is a set of indicators for time relative to the app adoption month, denoting *r* months before or after the month of adopting the health and fitness app; *γ_i_* is the individual fixed effects; *δ_t_* is the time fixed effects; and ε*_it_* is the error term. The coefficients of interest are *β_r_*’s, capturing the dynamic effects (the period *r* treatment effects) of the health and fitness app adoption on hospital visits over time.

The identifying assumption is that without the adoption of the health and fitness app, the outcomes of the treatment and control groups would run in parallel. The plausibility of our design depends on the notion that the adoption timing is as good as a random assignment. Although our strategy ensures that the experimental groups are similarly likely to adopt the health and fitness app within a relatively short time window (ie, from April to September 2019), there still are threats to credible causal inference.

First, in our empirical setting, the adoption of a health and fitness app does not mean that people would actually use the app [49]. What would eventually affect people’s health and hospital visits is the extent to which they use the apps and the subsequent changes in their health behaviors. However, it is not uncommon for an individual to install a health and fitness app but never use it. The second threat is the potential nonrandom allocation of preadoption conditions [47]. Different cohorts of adopters may follow different trends before app adoption, which may result in a potential noncomparability of treatment and control groups. Third, users are likely to be exposed to other health shocks during the same period, which may lead to potential confounding effects. These threats can violate the parallel trends assumption and invalidate the causal inference.

To alleviate these concerns, we conducted a difference-in-difference-in-differences (DDD) analysis using a subgroup of people who installed but never used the health and fitness app as a baseline. Specifically, the users are divided into a *used group* (consisting of people who used the app after the adoption; the treatment subgroup in the DDD specification) and a *nonused group* (consisting of people who never used the app after the adoption; the control subgroup in the DDD specification). As the difference across subgroups lies only in the use after app adoption (ie, the other conditions are expected to be the same), the DDD specification can further remove biases in the DD estimator. In addition, the likelihood of an unobserved shock biasing the DDD estimators is low because such a shock must not only coincide with the timing of app adoption but also affect the subgroups differently. Therefore, the DDD estimators provide us with the causal effect of health and fitness app adoption on hospital visits without requiring the parallel trends assumption. The DDD specification is as follows:


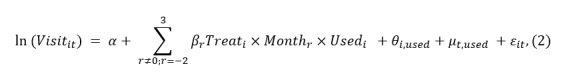


where *Used_i_* indicates whether a user use the app after adoption, *θ_i,used_* indicates the user-by-subgroup fixed effects, and *μ_t,used_* indicates the month-by-subgroup fixed effects [50].

## Results

### Descriptive Statistics

The number of unique users is 267,651 in our sample. Each user had 12 monthly records, resulting in a total of 3,211,812 observations. The average duration of hospital visits was 2.24 (SD 23.09) hours. The average number of days users were in the hospital per month was 0.43 (SD 2.22). On average, users used the focal app for 1.68 (SD 39.72) minutes per month. The average frequency of focal app use per month was 0.56 (SD 13.8). The average number of apps installed on the users’ phones in the adoption month was 35.65 (SD 29.79).

Although everyone in the sample adopted the app, only 21,444 used it (as in the used group in the DDD model). This means that many adopters did not actually use an app after its installation. Similarly, each user had 12 monthly records, resulting in 257,328 observations. For people in the used group, the average duration of hospital visits was 5.12 (SD 41.35) hours, and the average days of hospital visits was 0.74 (SD 3.22) days. This shows that people who used the app after adoption are more likely to visit the medical facilities. On average, people in the used group used the app 6.94 (SD 48.31) times per month, and the average duration of focal app use was 21.01 (SD 137.25) minutes. The average number of installed apps in the adoption month was 34.45 (SD 23.22).

**Figure 2:**
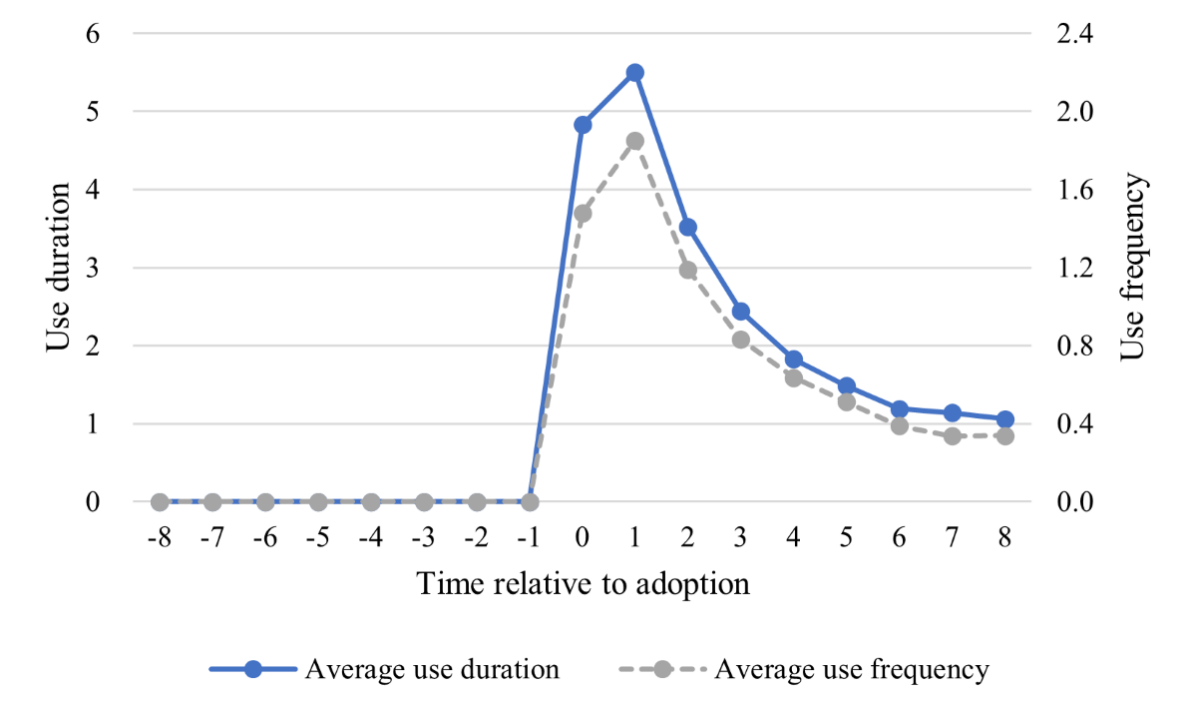
Average monthly use before and after the app adoption for all adopters.

**Figure 3:**
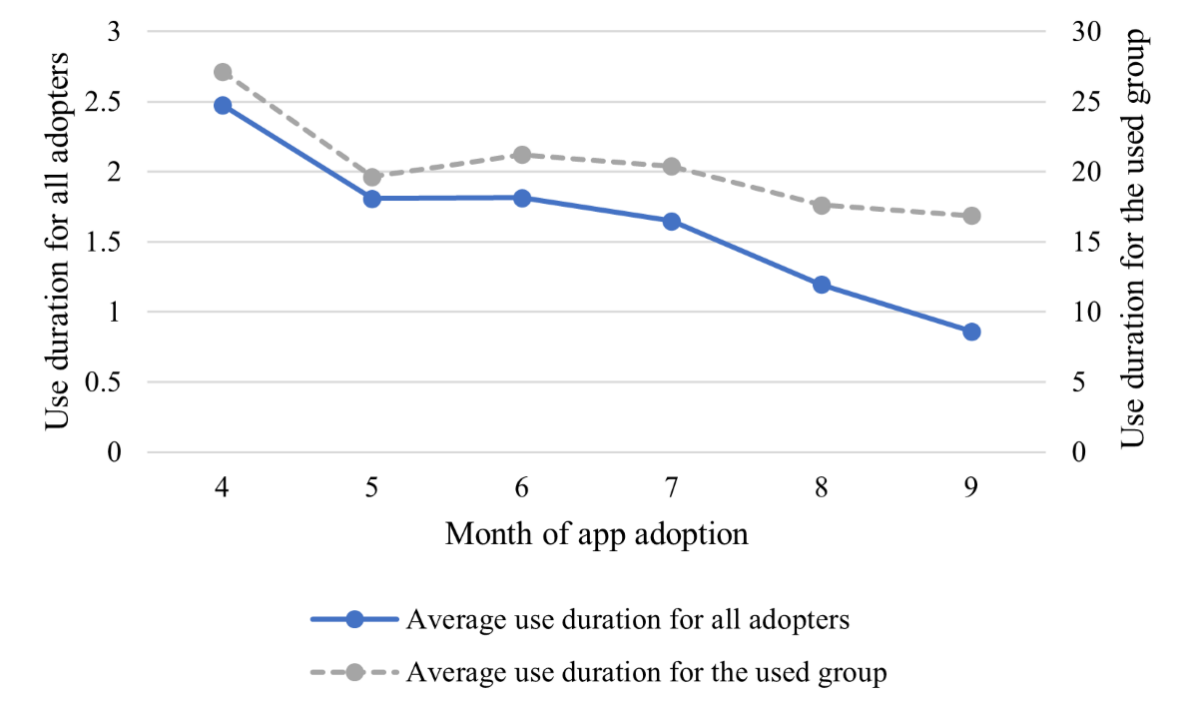
Average app use by month.

We next present further results. First, the average app use for all adopters before and after adopting the app is shown in Figure 2, where the left y-axis shows the duration of use (in minutes) and the right y-axis shows the frequency of use. A sharp rise in app use can be observed after users adopt the app, but app use declined substantially over several months. This is consistent with the high dropout rate for mobile apps. We then plotted average use over time in Figure 3, where the left y-axis shows the average duration of app use (in minutes) for all adopters and the right y-axis shows the average duration of app use for the used group. There was no significant difference among users who adopted the app in different months, indicating that users who adopted the app in different months were comparable. This ensured the validity of our identification strategy.

### Main Results

**Figure 4:**
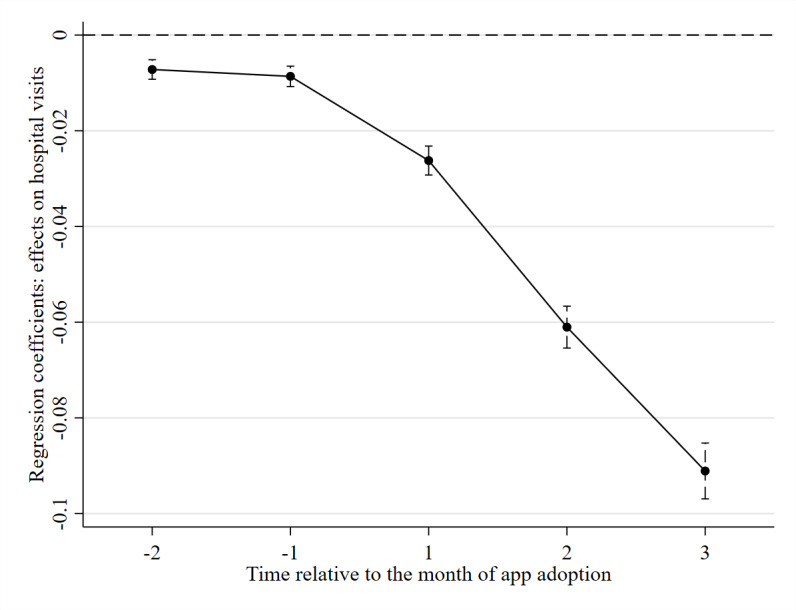
Dynamic treatment effects from baseline difference-in-differences specification.

The results of the baseline specification, which shows the dynamic effects of health and fitness app adoption on hospital visits, are reported in Table 1. We also plotted the dynamic effects with their 95% CIs, as shown in Figure 4. We found that, after the adoption of the health and fitness app, there was a substantial drop in the duration of hospital visits. Specifically, app adoption was associated with a 2.6% reduction (*P*<.001) one month after the adoption month, a 6.1% reduction (*P*<.001) two months after the adoption, and a 9.1% reduction (*P*<.001) three months after the adoption in the duration of hospital visits.

**Table 1:** Dynamic effects of health and fitness app adoption from baseline difference-in-differences specification^a^.

Time relative to adoption^b^	β (SE^c^)	*P* value
−2	−.007 (.001)	<.001
−1	−.009 (.001)	<.001
1	−.026 (.002)	<.001
2	−.061 (.002)	<.001
3	−.091 (.003)	<.001

^a^This table reports dynamic difference-in-differences estimates using baseline specification (equation 1). User and time fixed effects are included as controls (n=1,873,557; *R*^2^=0.609).

^b^It shows the effects in the months before and after app adoption.

^c^Robust SEs are clustered at the user level.

The DD identification strategy requires the parallel trends assumption. It means that there should be no significant difference in the trends of outcomes between the treatment and control groups before adoption [47], that is, *β_r_*=0 for all *r*<0. However, the preadoption effects are significant. Specifically, app adoption was associated with a 0.9% reduction (*P*<.001) one month before the adoption and a 0.7% reduction (*P*<.001) two months before the adoption in the duration of hospital visits. This suggests that the parallel trends assumption may not hold. We further performed a DDD estimation, which does not require the parallel trends assumption.

**Figure 5:**
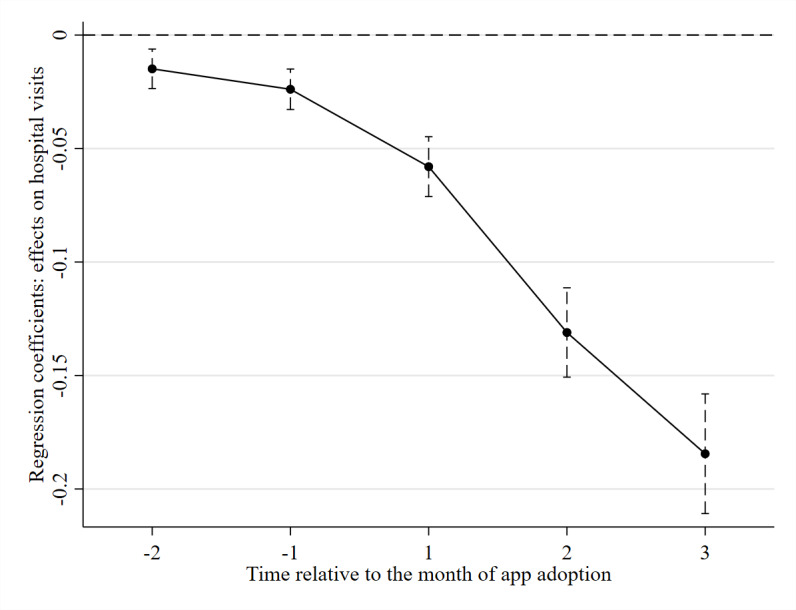
Dynamic treatment effects from difference-in-difference-in-differences specification.

The results of the DDD estimation are reported in Table 2, and the dynamic effects are plotted in Figure 5. The results again showed a significant decrease in the duration of hospital visits after app adoption. Specifically, app adoption led to a 5.8% reduction (*P*<.001) one month after adoption, a 13.1% reduction (*P*<.001) two months after adoption, and an 18.4% reduction (*P*<.001) three months after adoption in the duration of hospital visits. Compared with the baseline DD estimates, the coefficients of the DDD estimates are larger, indicating that some unobservable factors lead the baseline specification to underestimate the coefficients.

**Table 2:** Dynamic effects of health and fitness app adoption from difference-in-difference-in-differences specification^a^.

Time relative to adoption^b^	β (SE^c^)	*P* value
−2	−.015 (.004)	.001
−1	−.024 (.005)	<.001
1	−.058 (.007)	<.001
2	−.131 (.010)	<.001
3	−.184 (.013)	<.001

^a^This table reports the dynamic difference-in-difference-in-differences estimates using DDD specification (equation 2). User-by-subgroup effects and month-by-subgroup fixed effects were included (n=1,873,557; *R*^2^=0.609).

^b^It shows the effects in the months before and after app adoption.

^c^Robust SEs are clustered at the user level.

To verify that our findings are not limited to a specific time window, we shortened the app adoption time window to 4 months for both the treatment and control groups (ie, users who installed the app for the first time from May to August 2019 were selected) and ran the DD and DDD models in this shortened time frame. The results for the 2 specifications are not materially different (Multimedia Appendix 1).

### Sensitivity Analysis

In this section, we present our investigation on whether the results are robust to different measures of the dependent variable. To strengthen the robustness of our findings, we ran an analysis using alternative measures of hospital visits.

Specifically, we defined a hospital visit based on a user staying in a hospital for 3 and 4 consecutive epochs (as opposed to 2 epochs in the main analysis). We calculated the new versions of hospital visits and aggregated them into monthly data. This resulted in 2 alternative measures of hospital visits (one is based on 3 consecutive epochs and the other is based on 4 consecutive epochs). We used both to estimate the dynamic effects based on DD and DDD specifications. The results were not materially different from the main results (Multimedia Appendix 2). We also constructed another alternative dependent variable by counting the number of days when user *i* visits a hospital in month *t* during our sampling period. This measure alleviated measurement errors in hourly data. The results of both specifications were not qualitatively different from the main results (Multimedia Appendix 2). Therefore, our results were robust to how hospital visits are measured.

### Moderators

In this section, we explored the moderators that may influence the main effect. Prior studies have shown that individual demographics are associated with the use of health and fitness apps [51,52]. Thus, we examined the moderating effects of individual demographic factors, including socioeconomic status (eg, consumption level and city tier) and users’ digital literacy. The consumption level refers to a relative level of consumer spending (low, medium, and high levels). This variable is provided by the platform, which generates these data using a big data predictive model based on users’ mobile app use. Specifically, a consumption model was developed to predict each user’s consumption spending based on their app use characteristics, such as luxury shopping app use. A person’s spending behavior plays a crucial role in predicting their consumption patterns, and app use is an important determinant of the model. Users in the data set were then ranked based on the predicted consumption spending and classified into the low, medium, and high levels. The percentages of participants in the 3 groups were 40%, 40%, and 20%, respectively. The tier of the city where the users are located is defined based on the economic development of cities in China. Users’ digital literacy was measured by the total number of installed apps on the phone. We examined their moderating effects using the following specification:


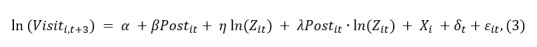


where *Z_it_* indicates the moderators of interest, including consumption level, city tier, and the total number of installed apps; *Post_it_* indicates whether user *i* adopted the app in month *t*; and *X_i_* indicates user fixed effects. The outcome variable is the duration of hospital visits for user *i* in month *t*+3 (ie, 3 periods after *t*) because there is evidence that a 3-month short-term exercise program may be sufficient to generate health benefits [53]. The results are presented in Table 3.

**Table 3:** Moderating effect analysis^a^.

Variables			Model 1: ln(Hospital visit duration)				Model 2: ln(Hospital visit duration)				Model 3: ln(Hospital visit duration)				Model 4: ln(Hospital visit duration)		
			Coefficient		*P* value		Coefficient		*P* value		Coefficient		*P* value		Coefficient		*P* value
Post			−0.030		<.001		−0.004		.57		0.037		<.001		−0.029		<.001
**Consumption level**																	
	Medium	0.029		<.001		0.035		<.001		0.029		<.001		0.029		<.001	
	High	0.186		<.001		0.234		<.001		0.186		<.001		0.186		<.001	
	Post*Medium	N/A^b^		N/A		−0.015		.03		N/A		N/A		N/A		N/A	
	Post*High	N/A		N/A		−0.106		<.001		N/A		N/A		N/A		N/A	
**City tier**																	
	Tier1	0.391		<.001		0.391		<.001		0.476		<.001		0.391		<.001	
	Tier2	0.377		<.001		0.377		<.001		0.453		<.001		0.377		<.001	
	Tier3	−0.017		<.001		−0.017		<.001		−0.016		<.001		−0.017		<.001	
	Post*Tier1	N/A		N/A		N/A		N/A		−0.190		<.001		N/A		N/A	
	Post*Tier2	N/A		N/A		N/A		N/A		−0.173		<.001		N/A		N/A	
	Post*Tier3	N/A		N/A		N/A		N/A		−0.002		.69		N/A		N/A	
**Total number of installed apps^c^**																	
	Appnum	0.017		.03		0.017		.03		0.016		.03		0.028		.001	
	Post*Appnum	N/A		N/A		N/A		N/A		N/A		N/A		−0.026		<.001	

^a^This table reports the moderating effects based on the specification (equation 3). The dependent variables of all models are hospital visit duration 3 months after the observation time *t*. Model 1 regresses the hospital visit duration 3 months after the observation time on the postadoption indicator and the set of individual characteristics. Models 2, 3, and 4 examine the moderating effects of consumption level, city tier, and the total number of installed apps, respectively. All models included user and month fixed effects. Robust SEs were clustered at the user level (n=172,494).

^b^N/A: not applicable.

^c^The total number of installed apps is mean centered.

The coefficients of the interactions are significantly negative (β=−.015, *P*=.03 for Post*Medium; β=−.106, *P*<.001 for Post*High) and the magnitude of the coefficient is much larger for the high level of consumption, showing that the higher the level of consumption, the greater the reduction effect of app adoption on hospital visits. This suggests that higher consumption–level groups (or higher-income groups) are more likely to benefit from health and fitness app adoption, possibly because higher economic levels amplify the health benefits of exercise and healthy lifestyles. An alternative explanation is that people with higher incomes are more likely to adopt unhealthy lifestyles (eg, sedentary behavior, physical inactivity, and high-intensity workload); therefore, they can gain greater health improvements from health apps.

Second, in high-tier cities, the reduction effect of app adoption on hospital visits was larger (β=−.002, *P*=.69 for Post*Tier3; β=.173; *P*<.001 for Post*Tier2; β=.190, *P*<.001 for Post*Tier1). In fact, we observed that the main effect is negatively significant in tier 1 and tier 2 cities but is positive in tier 3 and tier 4 cities (ie, the adoption of health and fitness apps will instead increase hospital visits for users in lower-tier cities). Adopting health and fitness apps may not be sufficient for people in lower-tier cities to obtain adequate health benefits or reduce their hospital visits, possibly because of their weaker health awareness and unhealthy lifestyle. It is also likely to increase their health awareness and thus encourage users to conduct more health screenings in hospitals. An alternative explanation is that people in lower-tier cities may lack exercise-related knowledge, instruction, or supervision, making them more prone to injury and thus requiring medical attention.

Finally, we investigated an individual characteristic, users’ digital literacy—that is, their ability to use digital or web-based products. We measured the factor based on the number of apps installed on an individual’s phone by the end of March 2019 (before the treatment window in our sample). The results (model 4) showed that adopting a health and fitness app leads to a larger reduction in hospital visits for users with higher digital literacy (β=−.026; *P*<.001 for Post*Appnum).

Model 1 regresses the duration of hospital visits 3 months after the observation time *t* on the postadoption indicator, controlling for users’ observable characteristics (ie, age, gender, consumption level, city tier, and the total number of installed apps) instead of user fixed effects. The result showed a 3% decrease (*P*<.001) in hospital visits. This analysis served as a robustness check for the main finding.

The results of model 2 showed that, compared with users with low consumption levels, users with medium consumption levels visited hospitals more (β=.035; *P*<.001), and users with high consumption levels visited hospitals even more (β=.234; *P*<.001). Users with higher consumption levels visit hospitals more often, likely because higher consumption levels are associated with higher incomes, and people with higher incomes are likely to be more health conscious, pay more attention to their health conditions, and adhere to health professionals’ recommendations to a greater degree. They are likely to invest more in both preventive and curative care, such as conducting health screenings and treating any illnesses. In contrast, people with lower consumption levels are more likely to ignore or adopt a laissez-faire attitude toward minor health problems because of their disadvantaged financial conditions.

We turned our attention to another socioeconomic variable: the tier of the city where users are located. The city tier is also correlated with users’ socioeconomic status. As such, we expected it to exhibit moderating effects similar to those of the consumption level. The results of model 3 showed some interesting findings. First, similar to the consumption level, the higher the city tier, the more hospital visits users make. People in higher-tier cities are more likely to have high incomes so that they can spend more on health care. They are also generally more health conscious because of their high level of education. Furthermore, people in higher-tier cities have easy access to medical resources. This may explain why they are more likely to visit hospitals.

Third, the findings were similar for users in tier 1 and tier 2 cities. Overall, in tier 1 and tier 2 cities, people visit the hospitals more often, and the adoption of health and fitness apps can reduce hospital visits; however, in tier 3 and tier 4 cities, people make fewer hospital visits, and the app adoption increases hospital visits. This shows that there are important differences in health behaviors between cities at different levels of economic development.

## Discussion

### Principal Findings

mHealth and fitness apps have been receiving increasing attention as a potential solution for public hospital overload. They can help users monitor their own health behaviors, send regular reminders, and provide recommendations to increase their physical activity. Using a panel data set containing users’ hospital-related geolocation and focal app use data, this study used a DD strategy to identify the causal effect of health and fitness app adoption on users’ hospital visits. The results of the baseline model suggested that adopting the health and fitness app contributes to a reduction in the duration of hospital visits.

As this result may suffer from a violation of the parallel trends assumption, we further conducted a DDD estimate to alleviate this concern. The results confirmed our main findings regarding the reduction effect of health and fitness app adoption. We also performed a sensitivity analysis to establish the robustness of our results regarding how we measure hospital visits.

The heterogeneity analysis revealed that socioeconomic factors moderated the main effect. Specifically, people with higher consumption levels and living in higher-tier cities make more hospital visits, and the adoption of the health and fitness app leads to a greater degree of reduction in hospital visits for these groups of people. This finding may be explained by the better population health awareness and medical facilities in higher-tier cities. In addition, we found an important difference between high-tier and low-tier cities: the effect of the health and fitness app is positive in low-tier cities. This is consistent with findings from the literature on the health disparities between rural and urban areas [54-56]. The findings suggest that although health-related information technologies have the potential to narrow the rural-urban health care gaps [57,58], they should be implemented with caution in lower-income areas because of the undesirable effects in these areas.

### Limitations

Our study had several limitations. First, this study investigated a key health outcome, hospital visit. Although the platform implements strict data quality control measures, the data may still have measurement errors. In addition, our data may not be fully representative of older adults, as they could potentially use mobile phones to a lesser degree than younger people. Unfortunately, an in-depth examination of the older adult cohort is not feasible, given that we lack precise information concerning their age. Future studies could address this issue by obtaining more actual data from hospitals or by conducting longitudinal field experiments. We also expected data that span larger observation windows to allow for more accurate analyses, but the outbreak of the COVID-19 pandemic hinders the use of data after 2020.

Second, although we estimated the causal effect of the adoption of health and fitness apps on hospital visits, we did not provide the mechanisms through which app adoption can decrease hospital visits. We suggested that adopting health and fitness apps can promote users’ engagement in physical activity, thereby contributing to better health outcomes and a decrease in hospital visits. For example, a recent field experiment showed that patients with diabetes who adopted an mHealth app participated in more exercises, which led to better health outcomes [59]. However, our data did not support the investigation of the underlying mechanisms.

Finally, people in the control group or the nonused group were likely to be exposed to the app and benefit from the app without using it. For example, people may be affected by other users who frequently use the app and the app may send promotional messages to encourage its use. This may lead to a spillover effect. This spillover effect would reduce the differences between the treatment and control groups (because a fraction of the users in the control group might also be exposed to the benefits of the app), thus leading to an underestimation of the effect of health and fitness app adoption. In the presence of the spillover effect, our estimates provided a lower bound for the true effect. Future studies can scrutinize the spillover effect by observing the social networks of app users and investigating peer influence.

### Theoretical and Practical Implications

Mobile apps have the potential to aggregate users’ informal health and fitness data and other relevant data (eg, hospital visits) to provide an integrated view for better health outcomes [60]. Prior work has primarily focused on identifying the critical factors that impact app use. We extended this stream of literature by investigating the outcomes of health and fitness app adoption. Our study also has implications for multiple stakeholders in the health care ecosystem.

First, this study empirically identified the causal effect of health and fitness app adoption on hospital visits. The observed link in some extant studies [28,29,31] may represent a mere correlation because time-varying unobservable factors may be correlated with the adoption of health and fitness apps and may confound the causal interpretation of the link. For example, health and fitness app adoption may be driven by people’s growing health consciousness or declining health status, which may also lead to fewer hospital visits. We used DD and DDD strategies to address identification challenges. We found that the adoption of health and fitness apps leads to a decrease in hospital visits. Our study represented a step toward using observational data for causal inference in the digital health literature.

Second, this study used a large data set with a diverse population to estimate the effect. Previous studies have typically used survey data or small-scale randomized experiments and focused on small populations [15,30-32]. This limited the generalizability of the findings. We used a data set covering multilevel groups of people; therefore, our findings may be generalized to the general Chinese population. More importantly, the integration of causal inference strategies and big data extends the research paradigm in the field of medical informatics from randomized controlled trials and surveys to observational data.

Third, we expanded the app adoption and use literature by examining the moderating effects of users’ socioeconomic factors and digital literacy. The findings on consumption levels and city tiers were also added to the literature on health disparities. We suggested that urban-rural health disparities should be considered in the research on health information technologies.

Our findings also have practical implications for health and fitness app users, medical service providers, health and fitness app developers, and policy makers. First, users may be uncertain about whether health and fitness apps are as effective as app developers claim. This study will help users make more informed decisions regarding whether they should use health and fitness apps. Second, our findings suggested that hospitals may advocate the use of health and fitness apps to help reduce overcrowding. Hospitals should encourage health professionals to recommend high-quality health and fitness apps to their patients. Third, app developers can benefit from our analyses of the moderating effects of users’ socioeconomic factors and digital literacy. They can develop personalized apps and features for different groups of people. For low-tier city users, priority should be given to raising their health awareness to prevent undesirable effects. Finally, our findings showed that health and fitness apps might be a viable solution to ease the burden on public health systems. Our study also provides insights into policies to reduce health care disparities. Policies should be customized for regions with different health care levels, such as establishing more health care facilities for lower-income cities to better leverage health-related information technologies.

### Conclusions

We found that the adoption of health and fitness apps reduces users’ hospital visits. The DD and DDD identification strategies estimate the causal effect. The heterogeneity analyses showed that the effect is greater for users with high consumption levels, in high-tier cities, and with high digital literacy. In contrast, for users in low-tier cities, the effect is positive (ie, health and fitness apps may actually increase hospital visits). Our study provides useful insights for multiple stakeholders in the health app and health care industry.

## Appendix

Multimedia Appendix 1 Results for a shorter observation window.

Multimedia Appendix 2 Sensitivity analysis.

## Abbreviations

DD: difference-in-differences
DDD: difference-in-difference-in-differences
mHealth: mobile health

## Data Availability

The data sets generated during and analyzed during this study are not publicly available because of the request of the platform and data privacy laws and regulations in China. Only aggregate data (eg, summary statistics) are available from the corresponding author on reasonable request.
