# Supplementary material for: The Effects of Adopting Mobile Health and Fitness Apps on Hospital Visits: Quasi-Experimental Study
Source: J Med Internet Res. 2023 Jul 28;25:e45681. doi: 10.2196/45681 (PMC10422177; doi:10.2196/45681)
Supplement: Multimedia Appendix 1 [file jmir_v25i1e45681_app1.pdf]

Multimedia Appendix 1: Results for models in the shortened time frame

Time relative to adoption	β	SE	*P* value
**Model 1: DD specification**[a]			
-2	-0.007	0.001	<.001
-1	-0.009	0.001	<.001
1	-0.027	0.002	<.001
2	-0.061	0.003	<.001
3	-0.092	0.004	<.001
**Model 2: DDD specification**[b]			
-2	-0.018	0.005	<.001
-1	-0.029	0.005	<.001
1	-0.051	0.008	<.001
2	-0.125	0.013	<.001
3	-0.177	0.017	<.001

[a]Model 1 reports dynamic difference-in-differences estimates using baseline specification (1) in the shortened time frame. User and time fixed effects are included as controls. N = 1,395,177, R-squared = 0.606. Robust standard errors are clustered at the user level.

[a]Model 2 reports dynamic difference-in-difference-in-differences estimates using DDD specification (2) in the shortened time frame. User and time fixed effects are included as controls. N = 1,395,177, R-squared = 0.606. Robust standard errors are clustered at the user level.
