# Supplementary material for: The Effects of Adopting Mobile Health and Fitness Apps on Hospital Visits: Quasi-Experimental Study
Source: J Med Internet Res. 2023 Jul 28;25:e45681. doi: 10.2196/45681 (PMC10422177; doi:10.2196/45681)
Supplement: Multimedia Appendix 2 [file jmir_v25i1e45681_app2.pdf]

Multimedia Appendix 2: Sensitivity Analysis

Time relative to adoption	*β*	SE	*P* value
**Model 1: Three consecutive epochs, DD specification (R-squared = 0.572)**			
-2	-0.005	0.001	<.001
-1	-0.006	0.001	<.001
1	-0.018	0.001	<.001
2	-0.043	0.002	<.001
3	-0.063	0.003	<.001
**Model 2: Three consecutive epochs, DDD specification (R-squared = 0.572)**			
-2	-0.014	0.004	.001
-1	-0.018	0.004	<.001
1	-0.048	0.007	<.001
2	-0.103	0.010	<.001
3	-0.145	0.013	<.001
**Model 3: Four consecutive epochs, DD specification (R-squared = 0.548)**			
-2	-0.004	0.001	<.001
-1	-0.004	0.001	<.001
1	-0.015	0.001	<.001
2	-0.032	0.002	<.001
3	-0.047	0.002	<.001
**Model 4: Four consecutive epochs, DDD specification (R-squared = 0.548)**			
-2	-0.011	0.004	.008
-1	-0.015	0.004	<.001
1	-0.040	0.006	<.001
2	-0.084	0.009	<.001
3	-0.111	0.012	<.001
**Model 5: Hospital visit days, DD specification (R-squared = 0.621)**			
-2	-0.005	0.001	<.001
-1	-0.006	0.001	<.001
1	-0.018	0.001	<.001
2	-0.041	0.001	<.001
3	-0.061	0.002	<.001
**Model 6: Hospital visit days, DDD specification (R-squared = 0.621)**			
-2	-0.008	0.003	.005
-1	-0.014	0.003	<.001
1	-0.036	0.004	<.001
2	-0.081	0.006	<.001
3	-0.116	0.008	<.001
